# Retention of ice-associated amphipods: possible consequences for an ice-free Arctic Ocean

**DOI:** 10.1098/rsbl.2012.0517

**Published:** 2012-09-12

**Authors:** J. Berge, Ø. Varpe, M. A. Moline, A. Wold, P. E. Renaud, M. Daase, S. Falk-Petersen

**Affiliations:** 1 University Centre in Svalbard, Pb 156, 9171 Longyearbyen, Norway; 2 Faculty of Biosciences, Fisheries and Economics, University of Tromsø, 9037 Tromsø, Norway; 3 Norwegian Polar Institute, Fram Centre for Climate and the Environment, 9296 Tromsø, Norway; 4 Akvaplan-niva., Fram Centre for Climate and the Environment, 9296 Tromsø, Norway; 5 School of Marine Science and Policy, University of Delaware, Lewes, DE 19958, USA

**Keywords:** Arctic, sea-ice fauna, conceptual model, deep sea, migration, life history

## Abstract

Recent studies predict that the Arctic Ocean will have ice-free summers within the next 30 years. This poses a significant challenge for the marine organisms associated with the Arctic sea ice, such as marine mammals and, not least, the ice-associated crustaceans generally considered to spend their entire life on the underside of the Arctic sea ice. Based upon unique samples collected within the Arctic Ocean during the polar night, we provide a new conceptual understanding of an intimate connection between these under-ice crustaceans and the deep Arctic Ocean currents. We suggest that downwards vertical migrations, followed by polewards transport in deep ocean currents, are an adaptive trait of ice fauna that both increases survival during ice-free periods of the year and enables re-colonization of sea ice when they ascend within the Arctic Ocean. From an evolutionary perspective, this may have been an adaptation allowing success in a seasonally ice-covered Arctic. Our findings may ultimately change the perception of ice fauna as a biota imminently threatened by the predicted disappearance of perennial sea ice.

## 1. Introduction

**Figure 1.**
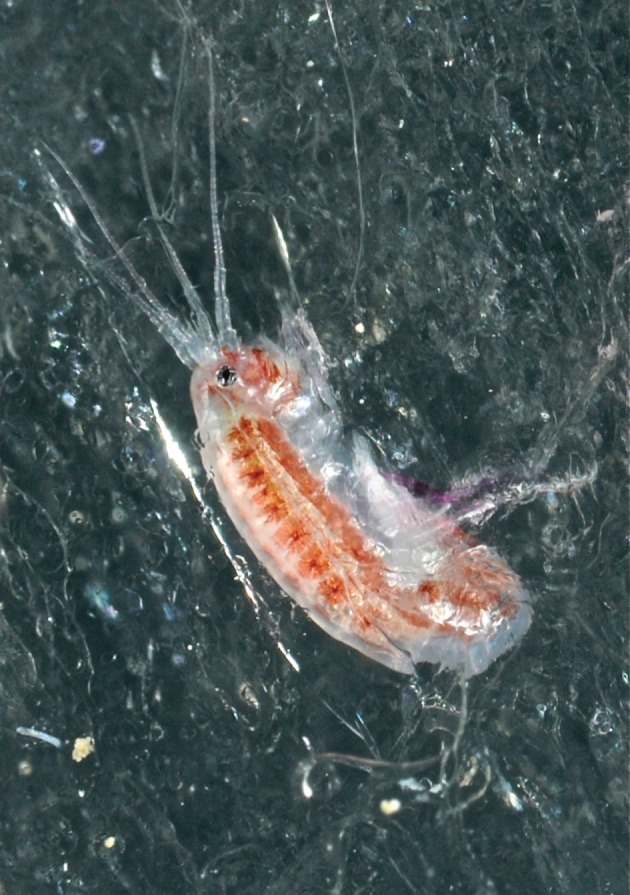
*In situ Apherusa glacialis* from sea-ice. Photo: Geir Johnsen.

The Arctic Ocean has been ice-free during the summer many times throughout the Quaternary Period [1], most recently only 8000 years ago [2]. Recent predictions suggest similar conditions within the next few decades [3]. This will be a significant challenge for all organisms dependent on the sea-ice habitat, particularly for those assumed to have an obligate association with the underside of the Arctic sea ice [4–7] and to complete their entire life cycle while directly connected to the ice [8–10]. More than 1000 taxa have been registered from Arctic sea-ice communities [11]. Amphipods are the most important in terms of biomass [6,12] and have long been recognized as central species in sea-ice ecosystems [13]. *Apherusa glacialis* (figure 1) is the most numerous of these ice-associated amphipods [12]. Based upon the collections from the Fletcher Ice Island T-3 expedition in the Arctic Ocean in the early 1950s and contemporary knowledge, it was considered to be a strictly pelagic amphipod [14]. More recently, this species has been recorded regularly, and in high numbers, attached to the underside of Arctic sea ice [9,10,12]. The scientific literature over the past 30–40 years [12] has regarded *A. glacialis* as one of the obligate ice-associated species feeding primarily on ice algae [9,12]. Nevertheless, the concept of obligate ice-associated organisms constitutes a paradox as to their survival during periods when their habitat has been absent, e.g. during periods of the Quaternary. Also, and perhaps more importantly, annual melting (68% reduction in 2011) [15], and export of ice (10–15% of the total ice-cover) [16] strongly diminish this habitat each year. However, ice amphipods are abundant in many areas with new first-year ice, often hundreds of kilometres away from the nearest location with any multiyear sea ice [12]. These observations suggest the existence of mechanisms that promote persistence of ice fauna in the Arctic Ocean.

## 2. Material and methods

All samples were collected using a multi plankton sampler (200 μm and opening of 0.25 m^2^) and a standard WP3 net (1000 μm and opening of 1 m^2^) onboard the *RV Helmer Hanssen* between 14 and 15 January 2012 at 81°45 N, 14° E. Water depth at location was 2200 m. *Apherusa glacialis* were present in all net hauls taken, but owing to challenging ice and wind conditions, only a total of four hauls were possible. The depth interval 0–200 m did not contain any individuals in any of the hauls. Haul 1 contained *A. glacialis* in depth intervals of 2000–600 m and 600–200 m (six and three individuals), haul 2 contained *A. glacialis* in depth intervals of 1200–600 m, 600–400 m and 400–200 m (three, one and one individuals), haul 3 contained *A. glacialis* in depth intervals of 1200–900 m and 900–600 m (four and one individuals). One additional net haul with a WP3 net contained *A. glacialis* (two individuals). The total lipid of individual specimens was extracted in chloroform: methanol following the method of Folch *et al*. [17], and total lipid measured gravimetrically.

## 3. Results and discussion

**Figure 2.**
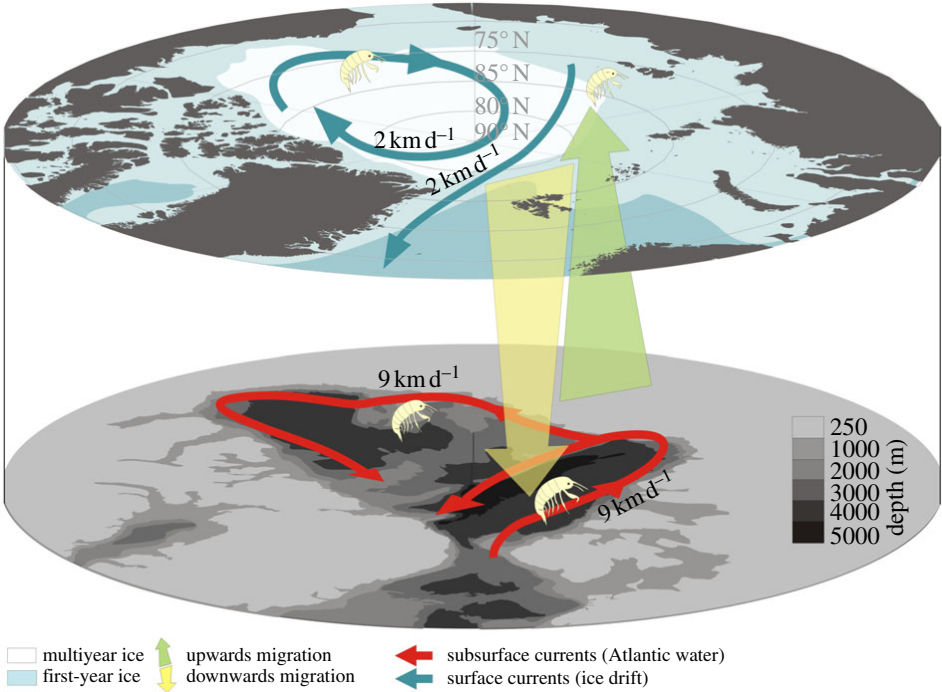
Conceptual model illustrating the intimate connection between ice-associated fauna and the deep Arctic Ocean currents. By performing deep migrations, organisms not only avoid export out of the Arctic Ocean, they are also actively being transported back into areas more likely to freeze early in the winter, and to areas where the expected lifetime of the ice is longer. This conceptual model also explains the otherwise unresolved paradox of how the obligate ice-associated fauna were able to survive warmer periods during the Quaternary Period without summer ice in the Arctic. The warm Atlantic current typically flows between 200 and 900 m depth within the Arctic Ocean, pictured only at 250 m on the figure.

A polar night campaign in the Arctic Ocean in early January 2012 provided a special opportunity to collect samples from a location and time of the year otherwise very poorly known [18–20]. Samples collected during this campaign revealed *A. glacialis* from depths between 200 and 2000 m (figure 2). Abundance estimates ranged from 16 to 36 ind. m^−2^ (see §2 for numbers per net), similar to those previously published from the Arctic sea ice [10,12]. Ovigerous females of this (and most) ice-obligate species have rarely been observed [9], but more than 50 per cent of the specimens collected were egg-carrying females, supporting the previously reported [9] winter reproduction of this species.

Based upon the oceanography of the Arctic and our unique, although limited, mid-winter observations, we propose a conceptual model (figure 2) that links the life cycle of *A. glacialis* with the deep Atlantic current flowing in the opposite direction from the transpolar ice drift within the Eurasian part of the Arctic. The model assumes a so-far undocumented ascent within the Arctic Ocean, but the well-documented recruitment of amphipods into newly frozen sea ice [12] provides at least circumstantial evidence for such an ascent. Deep water return currents (commonly 200–900 m; figure 2 [21]) are characteristic of the areas of ice export from the Arctic Ocean, particularly near the location of this study in the Fram Strait [16,21,22], but are also a dominating feature along the Arctic Ocean shelf break [21]. Accordingly, *A. glacialis* and potentially other ice fauna that migrate to the depth will be transported by these return currents against the drift of the pack ice from areas of their release from the melting ice back to the Arctic Ocean. This regional-scale, annual migration strategy is analogous to the diel/tidal migrations performed by other organisms to retain their positions in estuaries [23]. Also, it is comparable to the mechanism that has been described as part of the life cycle of *Calanus hyperboreus*, enabling it to retain its distribution within the central Arctic Ocean [22]. Finally, measurements of total lipid content (table 1) are comparable to that of *A. glacialis* collected from the ice [20] and for overwintering *Calanus* spp. [21], which perform both diel and seasonal vertical migrations, suggesting that energy stores are sufficient to complete the return passage to the central Arctic after their descent. In fact, other eusirids, and even *Apherusa* spp., have long been known to perform even extensive diel vertical migrations [24]. We therefore suggest that high-energy reserves combined with seasonal migrations are key elements of the annual routine of *Apherusa glacialis*, similar to many herbivorous zooplankton of high latitude oceans [25]. It should, however, be pointed out that our conceptual model rests upon a limited dataset, and our conclusions are yet to be rigorously tested.

**Table 1.** Lipid content of a subset of the collected specimens. Presence of eggs in brood pouch, length (L), dry weight (DW) and total lipid in percentage of DW (TL).

sex	egg	L (mm)	DW (mg)	TL
female	yes	9	2.5	64
female	yes	10	3.0	46.7
female	yes	11	4.0	47.5
female	yes	11	4.9	40.8
female	yes	8	1.5	66.7^a^
female	yes	8	1.2	66.7^a^
immature	—	7	1.0	46.7^a^
immature	—	7	1.0	46.7^a^
immature	—	6	0.9	57.7^a^
immature	—	6	1.0	57.7^a^
immature	—	6	0.7	57.7^a^
male	—	8	1.0	46.7

^a^Samples pooled according to size and sex.

*Apherusa glacialis* has previously been recorded from deep plankton nets during the polar night [14] within the Arctic Ocean, the Greenland Sea and the Fram Strait [7,26,27]. In common with interpretations of previous observations, this has not been seen as a potentially adaptive part of the life cycle, but as individuals passively exported away from melting sea-ice. Our conceptual model offers an explanation for these observations that goes beyond a passive sinking of organisms detached from their habitat. If these amphipods were totally dependent on sea-ice, the fact that the Arctic Ocean was most likely devoid of summer ice only 8000 years BP [2] suggests that the association must have evolved during a very short time period compared with the 60 Myr perspective associated with, for example, the evolution of Antarctic peracarids [28]. Rather, the annual reduction in sea ice by melting and export through the Fram Strait strongly diminish the habitat each year [15,16], and is a potential evolutionary driver that has been active at least throughout the entire Quaternary [1,2].

We argue that a pelagic phase may be an important part of *A. glacialis*’ life history and annual routine, developed as an adaptation to a seasonally ice-covered Arctic Ocean. In combination with the timing of reproduction, this behaviour both prevents export into the North Atlantic/Pacific [29] and enhances the opportunity for females to release their young in productive and ice-covered areas of the Arctic Ocean. Importantly, our proposed retention mechanism is not only relevant at the fringes of the Arctic Ocean. Even for amphipods positioned well within the Arctic Ocean, the same retention mechanism will be advantageous and typically return them to regions where export or lack of sea ice is even less likely.

From a food availability perspective, the polar night offers the best time for the organisms to detach from the sea-ice habitat. Mid-winter sea-ice holds a very limited amount of algal food; hence, the pelagic stage does not cause lost feeding opportunities. Furthermore, the very low light levels during winter cause low exposure to visual predators. The predation risk would therefore be low if the pelagic stage takes place during the polar night. Return-current speeds of 9 km per day [30] suggest that two to three months at depth is sufficient to counteract nine to 10 months of ice drift (2 km per day) [29]. Thus, provided that they stay in the deep during mid-winter, ice fauna can ascend and re-colonize the sea ice prior to the spring ice algal bloom. Although the specific adaptations and life-history traits differ for other species of ice-associated amphipods [31], our conceptual model and retention mechanism in areas dominated by sea ice may still apply. Hence, if our observations prove to be of a more general character, ice-associated fauna such as *A. glacialis* are less threatened by the predicted disappearance of Arctic summer sea ice than previously assumed. However, at the same time, it highlights the need for a more comprehensive understanding of processes governing the Arctic Ocean in general, and during the polar night, including energetic costs related to vertical migration during winter in particular.
